# Integrated Dataset of Screening Hits against Multiple Neglected Disease Pathogens

**DOI:** 10.1371/journal.pntd.0001412

**Published:** 2011-12-20

**Authors:** Solomon Nwaka, Dominique Besson, Bernadette Ramirez, Louis Maes, An Matheeussen, Quentin Bickle, Nuha R. Mansour, Fouad Yousif, Simon Townson, Suzanne Gokool, Fidelis Cho-Ngwa, Moses Samje, Shailja Misra-Bhattacharya, P. K. Murthy, Foluke Fakorede, Jean-Marc Paris, Clive Yeates, Robert Ridley, Wesley C. Van Voorhis, Timothy Geary

**Affiliations:** 1 Special Programme for Research and Training in Tropical Diseases, World Health Organization, Geneva, Switzerland; 2 African Network for Drugs and Diagnostics Innovation, United Nations Economic Commission for Africa, Addis Ababa, Ethiopia; 3 University of Antwerp, Antwerp, Belgium; 4 London School of Hygiene & Tropical Medicine, London, United Kingdom; 5 Theodor Bilharz Research Institute, Cairo, Egypt; 6 Northwick Park Institute for Medical Research, Harrow, United Kingdom; 7 University of Buea, Buea, Cameroon; 8 Central Drug Research Institute, Lucknow, India; 9 Ecole Nationale Supérieure de Chimie de Paris, Paris, France; 10 InPharma Consultancy, Herts, United Kingdom; 11 University of Washington, Seattle, Washington, United States of America; 12 McGill University, Montreal, Canada; Swiss Tropical and Public Health Institute, Switzerland

## Abstract

New chemical entities are desperately needed that overcome the limitations of existing drugs for neglected diseases. Screening a diverse library of 10,000 drug-like compounds against 7 neglected disease pathogens resulted in an integrated dataset of 744 hits. We discuss the prioritization of these hits for each pathogen and the strong correlation observed between compounds active against more than two pathogens and mammalian cell toxicity. Our work suggests that the efficiency of early drug discovery for neglected diseases can be enhanced through a collaborative, multi-pathogen approach.

## Author Summary

The search for new drugs for human neglected diseases accelerated in the past decade, based on the recognition that addressing these infections was necessary for global poverty reduction. The expansion of discovery and development programmes was supported by donor investment, increasing participation of the industry and the creation of Product Development Partnership (PDP) enterprises. Despite these efforts, major discovery gaps remain as, apart from some repurposed drugs and a few new molecules for malaria, no new candidate has been recently transitioned from discovery into development for the major Neglected Tropical Diseases (NTDs). In this publication, we present a collaborative network model for drug discovery based on coordinated North-South partnerships. This network carried out low-to-medium throughput whole-organism screening assays against seven NTDs (malaria, leishmaniasis, human African trypanosomiasis [HAT], Chagas' disease, schistosomiasis, onchocerciasis and lymphatic filariasis) together with an early assessment of compound toxicity in mammalian cells. We describe a screening campaign of 10,000 molecules, its outcome and the implications of this strategy for enhancing the efficiency and productivity of drug discovery for NTDs.

## Introduction

The search for new antiparasitic drugs for use in humans has accelerated in the past decade, based partly on the growing recognition that addressing these widespread infections is necessary for poverty reduction. There is a consensus that the drugs available for these pathogens are far from optimal, plagued by susceptibility to resistance, lack of activity against key species (or stages of the life cycle), lack of adequate efficacy in field-compatible delivery regimens, and reliant on single agents for control programmes [1], [2]. Expansion of programs for discovery and development of new compounds has been fueled by investment from donor organizations (such as the Bill and Melinda Gates Foundation and the Wellcome Trust) and increasing participation of the pharmaceutical industry. Some companies have established drug discovery centers for a select set of diseases. For example, the Novartis Institute in Singapore is focusing on malaria, dengue and tuberculosis and the GlaxoSmithKline (GSK) facility in Tres Cantos, Spain, is expanding its efforts to include a number of neglected diseases. We have also witnessed drug donation programmes essential for filariasis control by Merck and GSK as well as praziquantel donation by Merck Serono. This effort has been extended to sharing of proprietary and non-proprietary screening data, exemplified by the recent publication of screening results of corporate compound libraries against malaria parasites [3], [4]. In another development, Novo Nordisk transferred its entire compound library to the National Centre for Drug Screening in Shanghai to support drug discovery for neglected tropical diseases (NTDs) and related capacity building, in collaboration with The Special Programme for Research and Training in Tropical Diseases, at the World Health Organization (WHO/TDR) [5].

WHO-TDR has a long history of drug discovery and development for NTDs [1], [6], [7]. For several decades (1970–2000), it was the primary source of support for systematic antiparasitic screening programmes outside of military institutions and animal health companies [8], [9]. Since then, the creation and evolution of Product Development Partnership (PDP) organizations such as the Medicines for Malaria Venture (MMV), Drugs for Neglected Diseases initiative (DNDi) and Institute for One World Health (iOWH) to support development of promising drugs has become an important factor in bringing modern approaches to pharmaceutical research on neglected diseases [1], [10]. The development of sophisticated antiparasitic drug discovery activities in countries such as India, Brazil, South Africa and China, and increasingly in less developed countries in which these diseases are endemic, introduces new and influential contributors to the renaissance in this area [11]. Furthermore, the continuing efforts of the animal health industry in antiparasitic discovery, particularly in the area of anthelmintics, are being incorporated into human discovery programs [12]; almost all available human anthelmintics were initially developed for use in veterinary settings.

Despite these efforts, major gaps in the discovery of new chemical entities for neglected diseases remain, and apart from some repurposed drugs and few new molecules for malaria [13], [14], [15], the international community has not been able to transition novel chemical entities from discovery into development in the past 15 years. An innovation gap has therefore been defined for the various phases of the drug discovery process [16]. Against this background, it is critical to invest in the discovery of new drugs that will contribute in the medium to long term control of these diseases.

A network model for drug discovery against multiple neglected diseases has been described as a suitable mechanism to overcome these challenges [1], [16]. This approach involves a coordinated North-South network of collaborators (from both public and private sectors) which operate low- to medium-throughput primary screens against target organisms, supported by medicinal chemistry, drug metabolism and pharmacokinetics resources to kick-start hit-to-lead and lead optimization programs. Over the years part of this screening network funded by TDR has systematically optimized throughput to enable the testing of hundreds to thousands of pre-selected chemicals in diverse collections against the following: *Plasmodium falciparum, Leishmania infantum, Trypanosoma brucei, Trypanosoma cruzi, Schistosoma mansoni, Onchocerca lienalis or O ochengi* (counterparts in cattle of the human pathogen, *Onchocerca volvulus*) and *Brugia malayi*. Higher-throughput methods have been developed for whole organism antimalarial and anti-*Chagas* screens, both in industrial and non-industrial settings [3], [4], [17]–[20]. However, the throughput capacities of whole-organism screens employing the other pathogens are generally low to medium, permitting parallel testing of tens or hundreds to a few thousand compounds per batch [21].

TDR has taken advantage of the availability of multiple assays available within its network to implement coordinated screening of highly triaged small-to-medium sized libraries against multiple organisms, combined with a rapid assessment of mammalian cell toxicity. Although no *in vitro* test is fully predictive of *in vivo* activity, the TDR strategy is generally accepted as standard practice in neglected diseases screens [22], [23]. Consistent with a virtual operation, library evaluation, management and sampling are coordinated with the support of external partners and consultants, who have extensive experience in medicinal chemistry related to drug discovery. These consultants help with prioritization of compound collections to be screened, analysis of the resultant screening hits thorough chemo-informatics analysis and review of available literature on the compound or series identified. Data evaluation, communication and decisions by the drug discovery network are supported by a readily accessible, centralized database in which biological and chemical results from distant sites are uploaded in close to real-time processes for analysis and searching [24]. The database provides chemoinformatics strategies enabling substructure searches to analyze and expand structure-activity relationships (SAR).

Here we present the outcome of screening a set of 10,000 compounds against seven (7) NTD pathogens in whole-organism assays through a collaborative network model. The implications of this approach, which we refer to as an integrated multi-pathogen screening strategy, for enhancing drug discovery productivity and efficiency for neglected diseases are discussed.

## Materials and Methods

### Chemical library

**Figure 1:**
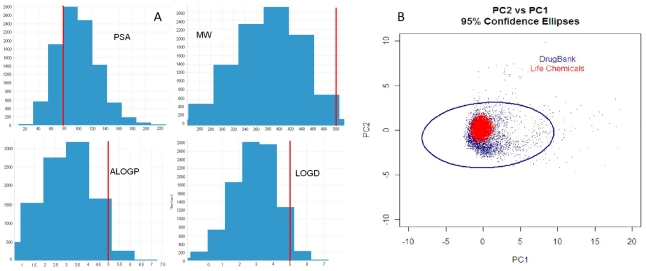
LifeChemicals library calculated properties analysis. A: Histograms representing calculated PSA, molecular weight (MW), aLogP and LogD for the selected Life Chemicals screening set. Overall, the library respects the criteria for the rule of five (maximum acceptable of each criteria represented by the vertical red line) except the PSA which has a slightly high calculated index. B: This grap is summarizing the Principal Component Analysis (PCA) of screened library versus PCA of DrugBank library. The DrugBank database is a resource that contains detailed data for about 6800 drugs. Calculated PCA show that the Life Chemical library (represented by the red ellipsis) sits within the 95% PCA confidence index of the DrugBank library (represented by the blue ellipsis). The PCA calculation was based on the descriptors to account for size, flexibility and polarity of molecules (molecular weight [size], number of rotatable bonds [flexibility], hydrogen bond acceptors [molecular polarity], hydrogen bond donors [molecular polarity], topological polar surface area [molecular polarity] and ALogP [molecular polarity].

A library of 10,000 compounds was purchased from Life Chemicals (Ukraine) after extensive review and analysis by medicinal chemists of over 50,000 available diverse structures. The key criteria used in the selection of molecules to be screened are diversity and novelty. To ensure a degree of diversity, selection was enhanced by using the computational informatics tool [Sybyl®, Tripos] [25]. Novelty of compounds for the target diseases was assessed by eliminating previously pursued compounds from in-house screens, available literature and comparison with chemistry already explored for neglected diseases. Based on this triage, a set of over 12,000 molecules was selected for further prioritization by analyzing for the presence of toxicophores and drug-likeness based on meeting at least 4 criteria in the Lipinski Rule of fives [26]. The confirmation by Life Chemical of the availability of close analogs for purchase to develop SAR, the simplicity of synthetic route and the availability in amounts >50 mg in the inventory, obviating the need for re-synthesis to permit testing in animals, was considered as well. The majority of the 10,000 compounds had a high degree of drug-likeness, in accordance with the Lipinski Rule of 5 (Figure 1). Compounds not fully compliant with the Lipinski Rules were included as a significant number of anti-infective and anti-cancer drugs do not meet the full criteria [27], [28]. Molecules were filtered to remove duplicate and reactive groups using Accelrys computational tools [29], to reach the 10,000 unique, non-reactive compound set. Life Chemicals provided the compounds with a special plate partitioning system to cover particular screening and logistics needs for multiple screening of this highly diverse compound set.

### Bioassay, screening logistics and framework

**Figure 2:**
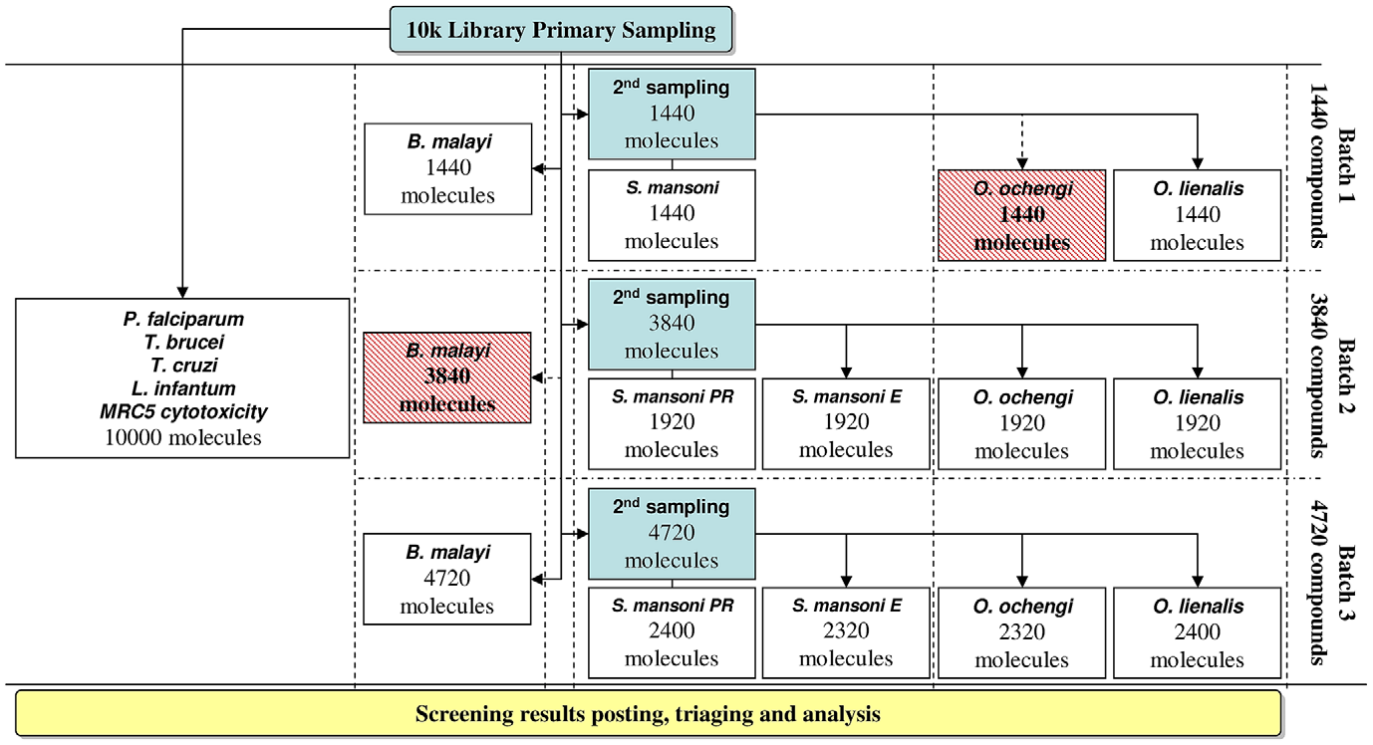
Screening logistics and framework. The formatting and distribution of the library to various screening centers on dry ice was handled by 2 screening partners - Laboratory for Microbiology, Parasitology and Hygiene at the University of Antwerp (LMPH) and the London School of hygiene and Tropical Medicine (LSHTM). Two shipments were lost (indicated by dark grey boxes) due to logistics problems. Considering the high variability of screening throughput (*P. falciparum* = 100,000 compounds/month, *T. cruzi*, *T. brucei & L. infantum* = 10,000 compounds/month, while *S. mansoni (adult worms), B. malayi (adult worms and microfilaria) and O. lienalis/O. ochengi (microfilaria)* = 1,000 compounds/month), shipments were handled in 3 batches from increasing size. For batch 2 and 3 and for diseases having 2 different screening sites (Schistosomiasis with London School of Hygiene and Tropical Medicine (LSHTM) and Theodor Bilharz Institute (TBRI) in Cairo or Onchocerciasis with Northwick Park Institute for Medical Research (NPIMR) using *O. lienalis* and the University of Buea (UB) using *O. ochengi*) it was decided to have molecules individually tested on one single site.

The compound screening logistics and framework, including sampling, distribution to various screening centers and screening cascade for the various parasites are shown on Figure 2. Standard Operating Procedures (SOPs) were implemented according to established criteria for advancement of compounds from primary screens to more advanced testing *in vitro* and for efficacy in animal models [16]. The integrated panel of antiprotozoal and anthelmintic screens used in the present study and the standard screening methodologies were adopted as previously described [9], [16], [30]–[35] (see Table 1).

**Table 1:** *In vitro* antiprotozoal [9], [16], [32]–[36] and anthelminthic screens [16], [30], [31], [37] screening protocols details.

A. Antiprotozoal screens	
For the antiprotozoan screens, stock solutions of each test compound were dissolved in 100% dimethyl sulphoxide (DMSO) at 20 mM and stored at 4°C until used. Medium throughput screens were performed in 96-well plates containing test compounds at 4-fold dilutions in a dose-titration range (64 µM to 0.25 µM). Each plate also had blanks, negative controls and reference controls with tests done in duplicate. For all protozoan strains studied, the selectivity index (SI) of each test compound was calculated from the ratio of the IC_50_ value determined in normal lung tissue (MRC-5) cells over the IC_50_ value determined from the dose-response curves (Statview™ software package) from each protozoa assayed.	
**Anti-** ***Plasmodium*** ** activity assay**	Parasites (*Plasmodium falciparum* K1 strain) were cultured at 37°C under a low oxygen atmosphere (3% O_2_, 4% CO_2_, and 93% N_2_) in RPMI-1640 supplemented with 10% human serum and human red blood cells (RBC). Two hundred microliters of infected RBC suspension (1% parasitemia, 2% hematocrit) were added to each well of the plates with test compounds and incubated for 72 hours. Parasite multiplication was measured by the Malstat method wherein 100 microliter of Malstat reagent were transferred in a new plate and mixed with 20 µL of the hemolysed parasite suspension for 15 minutes at room temperature. After addition of 20 µL NBT/PES solution and two hours incubation in the dark, the absorbance was spectrophotometrically read at 655 nm using a UV microplate reader. Percentage growth inhibition was calculated compared to the negative blanks. Test compounds with IC50 of <0.5 ug/ml and an SI of >100 were considered highly active.
**Antitrypanosomal activity assay**	Trypomastigotes of *Trypanosoma brucei brucei* Squib-427 strain were cultured at 37°C and 5% CO_2_ in Hirumi-9 medium (HMI) and supplemented with 10% FCS. The antitrypanosomal activity assay was performed by culturing 1.5×10^4^ trypomastigotes per well. Cultures were incubated with test compounds for 72 h at 37°C. After incubation, the growth of the parasites was measured fluorometrically (excitation wavelength of 530 nm and an emission detection of 590 nm) with the addition of resazurin to each well and incubated for an additional 24 h. Test compounds with IC50 of <0.5 ug/ml and an SI of >100 are considered highly active. *Trypanosoma cruzi*, Tulahuen CL2 strain, which are sensitive to treatment with nifurtimox, were maintained with MRC-5 cells in MEM supplemented with 20 mM l-glutamine, 16.5 mM sodium hydrogen carbonate and 5% FCS at 37°C and 5% CO_2_. The in vitro antitrypanosomal activity was studied by culturing 4×10^3^ MRC-5 cells with 4×10^4^ Trypanosoma cruzi parasites in each well, and then the wells were treated with each dilution of Pavetta crassipes extract. The culture plates were incubated for seven days at 37°C. The presence of parasite growth in each well was assessed by adding β-galactosidase and chlorophenol red β-d-galactopyranoside to each well and then incubating the plates for an additional 4 h at 37°C. The color reaction in the presence of parasites was measured at 540 nm, and the absorbance values were expressed as a percentage of parasites present in extract treated wells compared to the untreated controls. Test compounds with IC50 of <1 ug/ml and an SI of >50 were considered highly active.
**Anti-** ***Leishmania infantum*** ** activity assay.**	*Leishmania infantum* MHOM/ET/67 amastigotes were collected from an infected donor hamster and then used to infect primary peritoneal mouse macrophages. Test compounds were added to 3×10^4^ *Leishmania*-infected macrophages (seeded into each well of a 96-well plate). After 48 h, 5×10^4^ amastigotes were added to each well and then incubated for an additional 2 h at 37°C. Treated plates were incubated for 120 h at 37°C and 5% CO_2_. After the final incubation, each well was treated with Giemsa stain, and the number of parasites present in each well was counted under a microscope. The inhibition of parasite growth was expressed as the percentage of parasites in the compound treated wells versus the parasites in untreated controls. Test compounds with IC50 of <0.5 ug/ml and an SI of >20 were considered highly active.

B. Anthelminthic screens	
**Anti-** ***Onchocerca*** ** (** ***lienalis*** ** or ** ***ochengi*** **) and anti-** ***Brugia*** ** malayi activity assays**	A microfilariae screen was used initially as a higher throughput whole organism screen to select compounds for later testing against adult worms. Stock solutions of test compounds were prepared in 100% DMSO diluted into the medium. New compounds were tested using a 96-well format that contained a confluent feeder layer of monkey kidney cells. Compounds were tested at 12.5 uM or 10 uM for anti-*Onchocerca* (*lienalis* or *ochengi*) and anti-*Brugia malayi* activity assays, respectively. Culture medium was removed from the feeder layer and the test compound added followed by a transfer of 5 microfilariae onto each well. Worm viability was assessed using motility levels during a 5-day observation period. Test compounds with 100% inhibition of motility were considered highly active.
**Anti-** ***Schistosoma*** ** activity assay**	Stock solutions of 5 mg/ml of test compounds were prepared in 100% DMSO immediately before use. Test compounds were added to each culture well containing *Schistosoma mansoni* adult worms (5 each for male and female worms) in complete DMEM media) at a final concentration of 10 ug/ml or 12.5 µM. Worms were examined microscopically using an inverted microscope at intervals of 24, 48 and 120 h. Test compounds with 100% inhibition of motility were considered highly active.

Appropriate reference drugs, obtained either from Sigma or WHO-TDR collection, were used as positive controls [9], [16], [30]–[35]: chloroquine sulphate for *P. falciparum*, amphotericin B for *L. infantum*, nifurtimox for *T cruzi*, suramin and pentamidine for *T. brucei*, praziquantel for *S. mansoni*, immiticide, amocarzine and ivermectin for *O. lienalis* or *O. ochengi*, and ivermectin and diethylcarbamazine for *B. malayi*.

The compound set was tested against the following parasites: intra-erythrocytic forms in human red blood cells of *P. falciparum* K1 strain (malaria) [9], [16], [32], [33], [35], bloodstream forms of *T. brucei* Squib 427 strain (human African trypanosomiasis, HAT) [16], [31], [32], [34], intracellular amastigotes of *T. cruzi* Tulahuen CL2, beta- galactosidase strain (Chagas' Disease) [16], [32]–[35], intracellular amastigotes of *L. infantum* MHOM/MA(BE)/67 (leishmaniasis) [16], [32]–[36], larval and/or adult worms of *S. mansoni* Puerto Rican strain (schistosomiasis) [16], [31], [37], microfilariae of *B. malayi* (lymphatic filariasis) [16], [30] and microfilariae of *O. ochengi* or *O. lienalis* (onchocerciasis) [16], [30]. It should be noted that the human pathogen for onchocerciasis is *Onchocerca volvulus*, but due to lack of a suitable in vitro assay or animal models for this pathogen, *O. ochengi and lienalis*, which are both animal pathogens, are used in the primary and secondary assays [16], [30].

In some cases, particularly for the antifilarial screens, a second whole organism test with adult stage parasites is included prior to animal evaluation. The decision to utilize microfilariae for primary screening was based on throughput and biological considerations. It is difficult to acquire enough adult filariae to enable screening more than 1000 compounds per year [21], a rate far lower than needed for timely evaluation of the available compounds. In light of this situation, and considering that we are targeting molecules that are active on both stages, microfilariae screening was adopted as a suitable filter to identify compounds with potential macrofilaricidal activity [30].

An important part of the screen is the evaluation of all compounds for cytotoxicity using the diploid human embryonic lung fibroblast MRC-5 cell line (Figure 2). Further evaluation of cytotoxicity also takes advantage of the assays for intracellular protozoan parasites, as in the case of *L. infantum* and *T. cruzi*, which include murine peritoneal macrophage host cells [36] and MRC-5_SV2_ human lung fibroblast cells, respectively, in the culture system.

### Analysis of screening results

Hits identified from the screens were initially compared with cytotoxicity data for selectivity. Further filtering of the data was done using the published ‘Hit’ criteria for all pathogens combined with classical medicinal chemistry criteria [16]. All screening actives were evaluated for prior art through searches of Chemical Abstracts, and any relationship to compounds in development in related or other therapeutic areas investigated by substructure searches in drugs databases, principally the Investigational Drugs database (IDdb3; now Thomson Reuters Partnering). Substructure searches were also done in the TDR database covering previously tested libraries and medicinal chemistry projects.

## Results

### Screening results from seven pathogens

**Figure 3:**
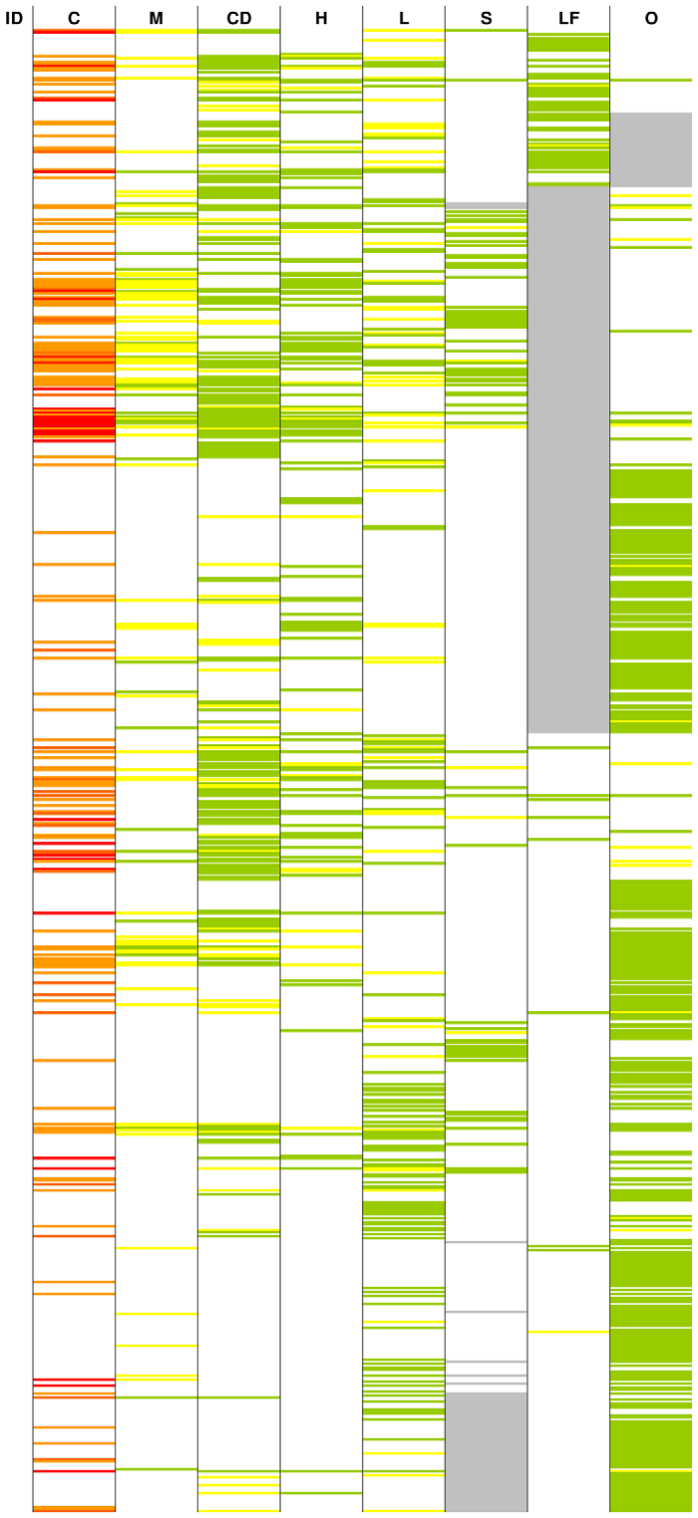
Comparison of the 744 hits from different pathogens and cytotoxicity. The 744 hits identified on the various pathogens are sorted by compound identifier (ID). Each colored line represents an active molecule and each column represents an assay for a pathogen or cytotoxicity (C = cytotoxicity, M = malaria, H = HAT, CD = Chagas disease, L = Leishmaniasis, S = Schistosomiasis, LF = Lymphatic Filariasis (microfilaria) and O = Onchocerciasis (microfilaria). Except for cytotoxicity, where active molecules are highlighted in red (activity<1 µg/ml), orange (1 µg/ml<activity<2 µg/ml) or gold ((2 µg/ml<activity<5 µg/ml) depending on the degree of activity, all molecules meeting the activity criteria for a disease pathogen (see Results) are highlighted in green and molecules having some borderline activity (Malaria and HAT: 0.5 µg/ml<activity<2 µg/ml, Chagas Disease: 1 µg/ml<activity<4 µg/ml, Leishmaniasis: 2 µg/ml<activity<4 µg/ml and Schistosomiasis/Onchocerciasis/Lymphatic Filariasis: >75% motility reduction at 12.5 µM) are highlighted in yellow. Grey box represent missing results. Further details on color coding are available below.

From the screen of 10,000 compounds, 744 were identified as active against at least one pathogen (Figure 3). Cytotoxicity data were used to aid selection and prioritization of compounds for progression to the next stage of the lead discovery process. To the best of our knowledge, this is the first time in which comparative data from essentially simultaneous screens against multiple neglected diseases pathogens and cytotoxicity data have been used in an integrated analysis for lead identification. Specific hit series from each pathogen are described below. To further illustrate the results from this multi-pathogen and cytotoxicity screening, a series of case examples with chemical structures covering one or more diseases are presented (Table S1). Identified hits are either referred to as series, consisting of a group of active molecules having similar core chemical structures/scaffolds, or as singletons if only one member of a chemical family is identified as active against a pathogen.

#### *P. falciparum* (Malaria)

33 of the 744 active compounds met the potency criteria of IC_50_<0.5 µg/ml for *P. falciparum* [16]. Further evaluation of these molecules resulted in the prioritization of 7 compounds, encompassed in 2 series and 2 singletons. One series, comprised of 2 molecules (not shown), is structurally related to a previously identified chemical series which is being optimized by an external partner (Merck Serono), providing an important validation of the library and the concept. The remaining series [structure # 8] with IC_50_ values of ∼0.15 µg/ml is structurally related to, but nonetheless distinct from, a series with activity against *T. cruzi* and *T. brucei in vitro* [structure # 9, 10, 11]. One singleton [structure # 15] is structurally related to a series with activity against *L. infantum* [structure # 38] and the other was distinguished by a notable lack of activity with close analogs tested in this assay and therefore is of interest [structure # 16].

#### *T. brucei* (HAT)

**Figure 4:**
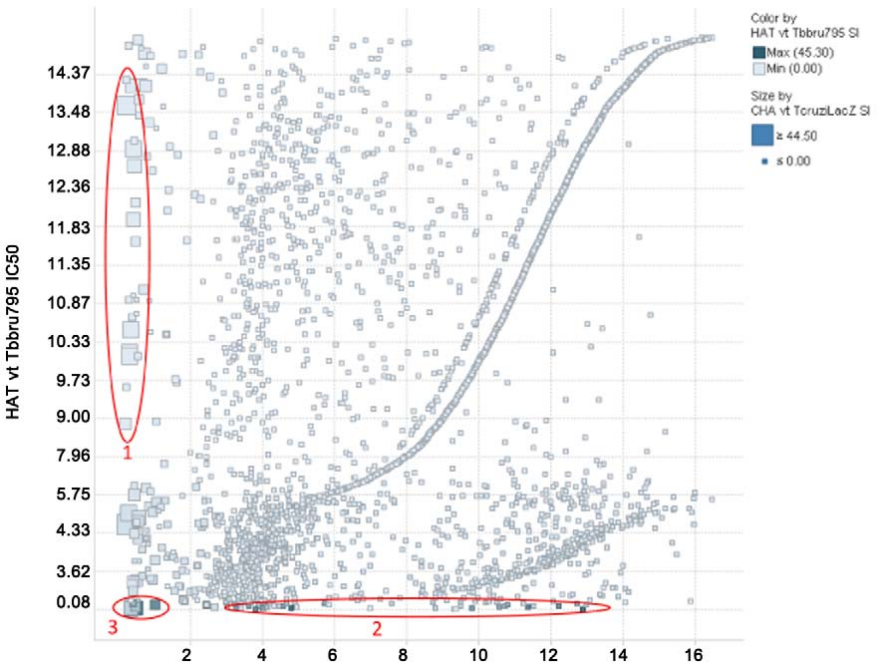
Comparison of Chagas (*T. cruzi*) and HAT (*T. brucei*) hits that are non cytotoxic. Graph show IC_50_ values of all non cytotoxic compounds (IC_50_>5 ug/ml on MRC5 cells) on Chagas (X axes) and on HAT (Y axes). Interesting series are grouped into 3 sub groups as follows: 1) the compounds that are active against only *T. cruzi* are represented by large light blue squares around the Y axes, 2) the compounds active against only *T. brucei* are represented by small dark blue squares around the X axes, and 3) compounds active against both pathogens identified as large dark blue close to the cross axes. The compounds in group 3 are potential candidates for joint development for the 2 diseases.

108 of the 744 actives passed the initial criterion of IC_50_<0.5 µg/ml for bloodstream forms of *T. brucei* Squib 427 strain. Of these, 67 compounds exemplified in [structures #1 to 6] were removed from further consideration due to poor selectivity vis-à-vis mammalian cells. An additional 7 compounds were discarded based on undesirable chemical attributes [structure # 39]. Of the remaining 31 molecules, TDR84116 [structure #37; proposed for evaluation at Merck Serono], showed exceptional potency (IC_50_ = 0.08 µg/ml) against *T. brucei* and was later reconfirmed with a similar potency (0.02 µg/ml, SI>100) against *T. brucei rhodesiense* (STIB-900). The remaining 20 actives represent 6 structural series and 11 singletons. All were less potent than TDR84116, with many showing IC_50_ values near the cut-off point. Some of these series are also active against *T. cruzi* [structure #7 and Figure 4).

One series and two singletons were prioritized for further investigations [structures #17, 18 and #19, 20, respectively]. One series was prioritized for the onchocerciasis program (data not shown), while a third series appears interesting for a joint HAT/Chagas project [see structures # 11, 12 and Figure 4].

#### *T. cruzi* (Chagas' Disease)

171 of the 744 actives passed the initial criterion of IC_50_<1 µg/ml for *T. cruzi* Tulahuen CL2, beta galactosidase strain maintained on MRC-5_SV2_ cells in MEM medium (MRC-5 cell/parasite inoculum: 4.10^3^ cells/well+4.10^4^ parasites/well), supplemented with 200 mM L-glutamine, 16.5 mM NaHCO_3_ and 5% heat inactivated fetal calf serum. Not sure that the details of the culture system and medium belong here. Of these, 109 were eliminated [structures #1–6] for poor selectivity versus mammalian cells (SI<50) and an additional 11 due to undesirable chemical attributes. The remaining 51 compounds were sorted into 10 series (45 compounds) and 6 singletons [structures # 21, 22]. Two of these series with excellent potency against *T. cruzi*, limited activity against other protozoa and SI values >50 (not shown), have been transferred to DNDi for further evaluation.

A series of 2-aminothiazole derivatives [structure # 23] was transferred to the University of Sao Paolo for further exploration, though potency and selectivity were lower than those observed for the series transferred to DNDi. A series with a benzo(thiazol-2-yl)acetamide pharmacophore [structure # 9, 10, 24] included 14 molecules with activity at concentrations as low as 0.22 µg/ml with an interesting pattern of selectivity against other pathogens. However, some showed limited selectivity against mammalian cells. Additional investment of medicinal chemistry should be made to clarify the potential for selectivity among the various parasites and especially with regard to mammalian cells, prior to analysis *in vivo*.

#### *L. infantum* (leishmaniasis)

115 compounds met the criterion of IC_50_<2 µg/ml against *L. infantum* amastigotes in macrophages. Of these, 30 [structure # 25] were discarded for poor selectivity against mammalian cells (SI<20) and another 8 for undesirable chemical attributes [structures # 1, 2, 5, 26]. The remaining 77 compounds were organized in 11 series (58 compounds) and 22 singletons. In addition to the series described above for *P. falciparum* [structure # 16], several series are considered high priority based on potency (IC_50_<0.5 µg/ml in ≥2 compounds) and SI values >30, as well as on preliminary SAR and chemical considerations. One series [structure # 36, 40, 41], includes 17 molecules with no previous evidence of antiparasitic applications and, for most of the molecules, a clean selectivity profile against tested pathogens. Two other high interest series with respectively 9 and 6 analogues are represented by structures #42 and #43.

#### *S. mansoni* (schistosomiasis)

A subset of 9478 of the 10,000 compound collection was tested against the Puerto Rican strain of *S. mansoni*. 6144 of these were tested in a primary larval assay [37] followed by the ex-vivo adult secondary screen. The remainder was screened only in the adult assay. 65 of the 744 actives from all pathogens met the minimum criteria for activity against adult *S. mansoni*: (i.e., 100% reduction in worm motility at a concentration of 10 µg/ml or 12.5 µM). The 65 compounds were sorted by chemical attractiveness, potency against adult schistosomes in culture, selectivity against other parasites, and toxicity to mammalian cells in culture. Based on this analysis, 5 series encompassing 13 compounds were chosen for advancement through the African Network for Drug and Diagnostic Innovation (ANDI) [11] and other PDPs. Representative structures [structures # 13, 27, 28] are shown (Table S1). These compounds are unburdened by obvious toxic moieties, have acceptable compliance with the Lipinski rules, are selectively active against schistosomes and are not overtly toxic to mammalian cells. It has unfortunately not been possible to titrate all of these hits in culture; of about 50% for which such data are available, none has an IC_50_<2 µg/ml (which is the initial potency criteria). Further analysis of these series, including confirmation, titration, testing of additional analogs and assays of *in vivo* activity in mouse models of schistosomiasis, is needed to prove that any has sufficient merit to warrant further development.

#### *Brugia malayi* (lymphatic filariasis)

As result of logistics issues attributed to lost shipments of some compounds, only 6160 molecules of the 10,000 compound set were tested, at a uniform molarity [30], against *B. malayi*, including 6063 against microfilariae and 756 against adult worms (these sets overlap). Activity criteria were 100% loss of motility at 10 µM for either stage. Greatest priority was given to compounds with activity against adult worms, as no macrofilaricide is available for use in control campaigns and the activity of ivermectin as a microfilaricidal agent is unlikely to be bettered in a new series. Of the test set, 53 compounds met the activity criterion for microfilariae (100% motility reduction) and an additional 58 inhibited adult worm motility ≥75% at 10 µM. These were titrated to determine an IC_50_ value to support prioritization. Following cytotoxicity triage [structures # 1, 4, 6], eight compounds met the activity criterion [16] for adult worms (i.e., 100% inhibition of adult worm motility), four of which were also active against microfilariae [structures # 14, 29, 30, 32]. An additional 10 compounds with inhibition of motility ≥75% at 10 µM and an experimentally determined IC_50_ value were also considered. Of these, the most interesting was TDR76699 [structure # 29]; this compound has very good activity against adult worms (IC_50_ = 0.1 µg/ml) and is also active against microfilariae (IC_50_ = 0.7 µg/ml). None of the other compounds tested against adult parasites had IC_50_ values ≤0.5 µg/ml, a threshold which would reduce concerns about specificity. However, a few series [structures # 31, 33, 34], appear interesting for future medicinal chemistry projects.

#### *O. ochengi* or *O. lienalis* (onchocerciasis)

4240 compound s were screened against *O. ochengi* at the University of Buea in Cameroon, while a subset of 5088 compounds was successfully screened in UK by the Northwick Park Institute for Medical Research against *O. lienalis*, at a uniform molarity [30]. 7 compounds showed activity against microfilariae of *O. lienalis* and 330 were active against microfilaria of *O. ochengi* (100% inhibition of motility at 12.5 µM). After removing molecules that were toxic to mammalian cells (IC_50_<5 µg/ml), 292 molecules remained for further evaluation. It is interesting to note that of the 330 *O. ochengi* microfilaria positives, 48 have already been found to also have full to moderate activity on the adult worms. From the latter sub-set, 3 distinct chemical series and one singleton [structure # 35] were active against both microfilaria and adults. One series [structures # 2, 3] was deemed not interesting due to lack of selectivity against mammalian cells. The remaining series is being transferred to ANDI for further progression as part of a new collaboration.

In summary, of the hits that survived toxicity triage, at least four series covering malaria, schistosomiasis, Chagas' disease and human African trypanosomiasis have been transferred to external partners (Merck Serono, DNDi and the University of Sao Paolo) for further exploration of SAR, whilst promising series for leishmaniasis, schistosomiasis and onchocerciasis are being transferred to ANDI. Details, including structures of hits for malaria, HAT, Chagas' Disease and LF, that are not restricted by any legal agreements, are available through the TDR target database - www.tdrtargets.org [38], [39].

## Discussion

Historically, antiparasitic drug discovery has relied on whole organism screens, as exemplified by recent reports of promising leads from whole-malaria parasite screens [4], [13], [18]. Although progress has been made in drug discovery built on mechanism-based screening approaches and in identifying parasite proteins which are good drug targets, clear success stories are not yet available [38], [39]. Similar but much more limited screening efforts have been devoted to less publicized NTDs, including those caused by kinetoplastids and helminths. Whole parasite screening for drug lead identification for kinetoplastids has been limited, and outside of the animal health industry, few systematic efforts have been directed at screening against helminths. TDR efforts to identify novel starting points using whole-organism screening for the more neglected diseases warrant recognition and further support.

This work underscores the need for increased efforts in integrated screens for neglected diseases. The use of specific library selection methods which exclude previously described or known scaffolds and the early filtering of active molecules provides novel mechanisms to enhance screening outcomes for NTDs. A striking outcome of the current approach is the low number of actives identified for *P. falciparum*. This is due to the nature of the compound set and the fact that most known actives against *P. falciparum* were eliminated from the library. However, one of the identified novel malaria hit series is structurally similar to a previously identified series undergoing lead optimization in collaboration with Merck Serono (data not shown). This screening strategy also highlights the critical importance and value of early cytotoxicity assessment (Figure 3 and Table S1). This single test allowed us to rapidly discriminate highly attractive series from poorly selective molecules, as most cytotoxic compounds also hit multiple pathogens. It therefore helps to remove nearly all non-selective molecules, meaning molecules active against >2 unrelated pathogens [structures # 1 to 6]. It is noteworthy that we identified only one molecule [structure # 36], with a cytotoxicity IC_50_<5 µg/ml that was active against >2 pathogens.

The cost of resolving selectivity issues in secondary assays, or even later during clinical trials, can detract greatly from efficiency of the overall operation. We consider that lead series identified in *in vitro* assays, subjected to cross pathogen analysis as described in this paper, represent the most promising candidates for *in vivo* follow-up, minimizing resource wastage on unpromising hits. Although discovery is an expensive endeavor, expansion of chemistry or transition of a compound to development with associated ‘hit-to-lead’, lead optimization and preclinical toxicological assessment markedly escalates the investment. It is vital to advance only compounds that have a legitimate chance of success, especially with limited resources. Our approach of screening against multiple pathogens represents a path to overcoming some of the challenges of early drug discovery.

Our results also show that none of the anthelmintic hit series which showed no appreciable cytotoxicity was active on > one helminth pathogen, i.e., identified positives are selectively active in the schistosomiasis, onchocerciasis or LF screens [Table S1, structure # 27–35]. This again justifies the multiple species approach, as it helps to rapidly identify compounds that are active against multiple pathogens. Therefore, it helps to determine whether such overlapping activity is indicative of general toxicity as well as different or related mechanisms of action in different or related species, respectively. As indicated earlier, some hits against *T. cruzi* also showed activity against *T. brucei* [structure #11, 12 and Figure 4]. The activity shown against related kinetoplastid parasites (*T. brucei* and *T cruzi*) is suggestive of a specific mechanism of action. Although azoles may be active against different parasites, they probably act by different mechanisms in each [40]. The cross-Phylum activity of artemisinins is another example [41], but depending on the target product profile for the disease [1], [42], this kind of activity should be a signal for caution in pursuing a series. It should also be noted that a few non-cytotoxic hits against one or two protozoa are also active against one helminth (Table S1, structure # 30). Noteworthy are series in which some compounds display an activity against one species, whereas other compounds display activity against another species. The mechanism of action may be the same, but with a slight difference of target structure between the 2 parasites. In this case, the chemical exploration of SAR could be beneficial to research on both species.

The major benefit of the current approach centers on the synergy gained by simultaneously screening a compound collection against multiple organisms. Salient advantages include:

- Compound handling can be better coordinated and organized to gain efficiency in the distribution process.
- Interpretation and analysis of data can be done in a coordinated manner, so that decisions about compound progression can be integrated with programmatic priorities for the various indications.
- Areas of greatest need can be balanced with quality of hits (in both chemical and biological terms) to produce a portfolio of leads that offers the best chance for making significant advances in their respective area.
- It provides early validation of and confidence in hits identified and prioritized for the various pathogens.
- It helps to rapidly share lessons and create value around a set of compounds due to the significant amount of data collected from the multiple screens.

It should be emphasized that it has been traditionally difficult to generate novel, robust hits against leishmaniasis, leaving the current screening and drug discovery pipeline weak for this disease (www.dndi.org). It is therefore remarkable to see the interesting hit rate found in our screening campaign. The fact that we discovered different compound series, containing up to 17 similar molecules active against *L. infantum*, gives some degree of confidence to the results. This again points to the usefulness of careful selection of chemical libraries used for screening.

Presently, whole-organism screens for many of these parasites are not amenable to the scale of throughput possible with *P. falciparum* in erythrocytes [20]. However, the integrated screens against the 7 pathogens reported here demonstrate the feasibility and importance of leveraging resources across parasite screens. It can also aid the development of SAR data around identified starting points. In the context of the actives described here, expansion of a series should be accomplished by acquisition of close-in analogs such that hit series from the various screens can be further prioritized across indications. It would be interesting to evaluate the previously identified molecules from the various antimalarial screening projects using the approach described in this paper. This early evaluation could help to select the most promising series for development based on selectivity, thereby limiting the high risk inherent in transitioning molecules from early discovery to early development.

While the integrated screens presented here represent current “best practice”, many unknowns plague the screening pathways for new antiparasitic agents for human use. The dearth of safe and effective drugs for most NTDs means that it is difficult to fully validate the screening streams. Some of the most important antiparasitic drugs seem to require an interaction with the host immune system, for example, praziquantel for schistosomiasis and ivermectin for filariasis [2], a paradigm that is difficult to incorporate into a whole-organism screen in culture. We do not know how closely parasites in culture resemble parasites in a host in terms of drug target expression and the role of these targets in survival in the host. Some of the screens employ representative species for discovery, even though there are well known differences in sensitivity of other species in the same genus or clade (oxamniquine, for example, is active against *S. mansoni* but inactive against *S. haematobium* [42]. In some cases, the target parasite species of greatest interest is unavailable for screening (for example *Plasmodium vivax*, *Wuchereria bancrofti*, *Onchocerca volvulus*). Finally, the screens do not always employ the most important target stage of the parasite (microfilariae vs. adult filariae, for example). The screens are thus compromises between ideal and practical operations, with the central assumption inherent in this strategy: that drugs with broad-spectrum activity within a parasite group will be discovered and are of the greatest interest. On the logistics side: the network screening model requires careful plate formatting and repartitioning to minimize risks of inability to trace inter or intra screening variations.

### Conclusion and future perspectives

The integrated, multi-pathogen, collaborative approach to the discovery of new chemical entities described here promises to increase efficiency while reducing costs and second-stage attrition in the discovery of high-quality leads compared to operations which target pathogens individually. Although this work focuses on the initial whole organism pathogen screening programme for hit identification, it should be stressed that it is part of an integrated North-South drug discovery platform that includes medicinal chemistry, pharmacokinetics and early toxicology [1], [16]. The approach needs to be scaled up and implemented more broadly, especially in developing countries, to ensure that the diverse set of NTDs receives the attention they warrant. This will require enhanced financial support and commitment from stakeholders as the current funding level is grossly insufficient relative to the burden of these infections on the developing world. In this context, a concerted global effort that interfaces with and strengthens the R&D capacity of institutions in developing countries, for example through ANDI, would greatly maximize resources and ensure that resultant drug leads are efficiently advanced. In support of this approach, some of the hits described in this paper are being transferred to ANDI to support innovation and capacity building in Africa. In addition, the chemical structures for most of the hits from the different pathogens described in this paper can be accessed through the TDR targets database (www.tdrtargets.org), a global open source database of molecular targets and drug like compounds that supports open innovation for infectious tropical diseases [38], [39].

## Supporting Information

Table S1 Results table.
(DOC)
