# Supplementary material for: Integrated Dataset of Screening Hits against Multiple Neglected Disease Pathogens
Source: PLoS Negl Trop Dis. 2011 Dec 20;5(12):e1412. doi: 10.1371/journal.pntd.0001412 (PMC3243694; doi:10.1371/journal.pntd.0001412)
Supplement: Table S1 — Results table. (DOC) [file pntd.0001412.s001.doc]

Table S1: Results table

| ID # | STRUCTURE | TDR ID | CYT | MAL | CHA | HAT | LEI | SCH | LF/MF | LF/AW | ONC/MF | ONC/AW |
| --- | --- | --- | --- | --- | --- | --- | --- | --- | --- | --- | --- | --- |
|  |  |  | IC50 μg/ml | | | | | % Motility Reduction at 12.5μM | | | | % worm Mortality @ 12.5μM |
| 1 | 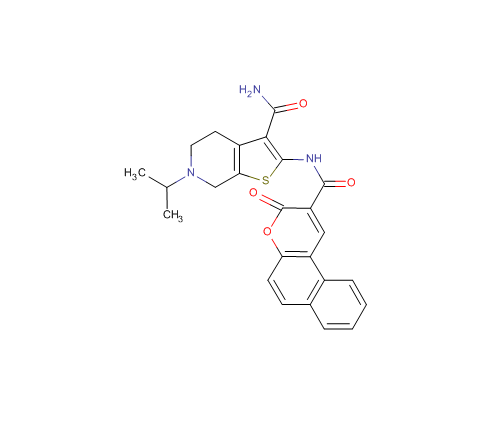 | TDR76885 | 0.42 | 0.57 | 0.15 | 0.48 | 0.48 | 0 | 100 | 100 | - | - |
| 2 | 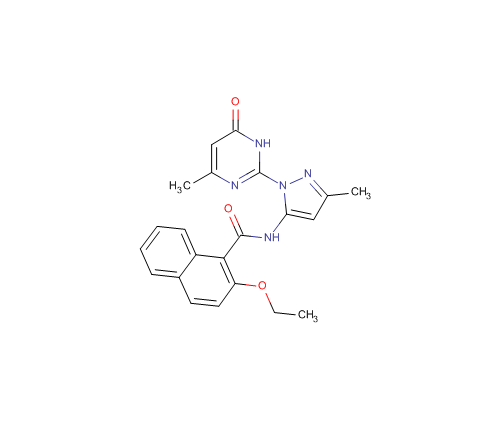 | TDR79930 | 0.36 | 0.4 | 0.12 | 0.33 | 0.63 | 100 | - | - | 100 | 60 |
| 3 | 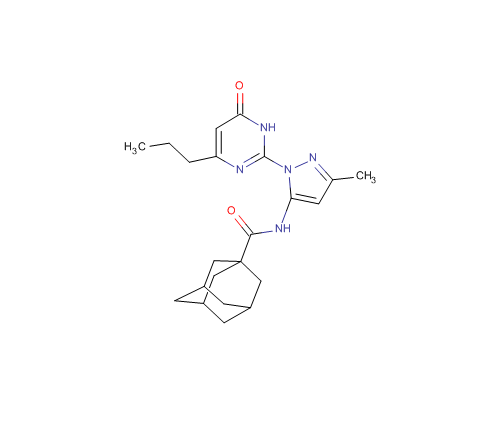 | TDR79936 | <0.12 | <0.12 | <0.12 | <0.12 | 3.84 | 100 | - | - | 100 | 75 |
| 4 | 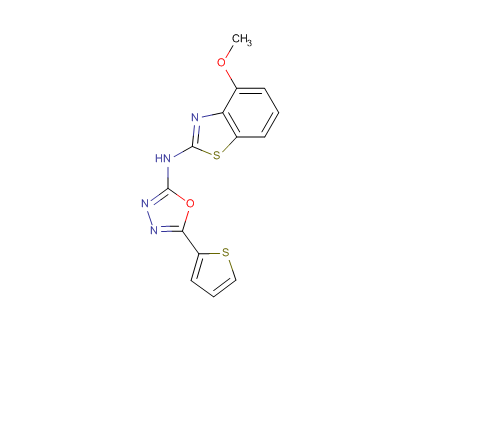 | TDR77585 | 1.83 | 1.25 | 0.31 | 0.35 | 3.17 | 0 | 100 | 50 | - | - |
| 5 | 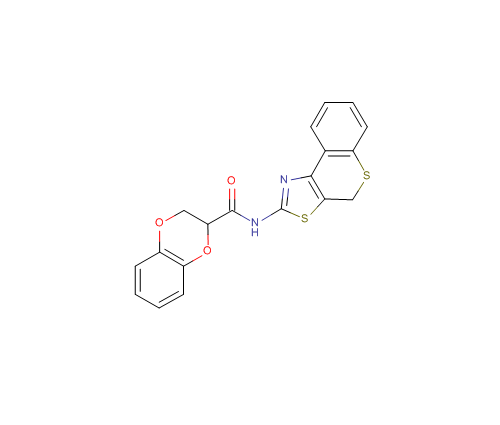 | TDR77035 | 2.62 | 4.75 | 0.5 | 0.37 | 0.78 | 100 | 33 | 0 | 100 | - |
| 6 | 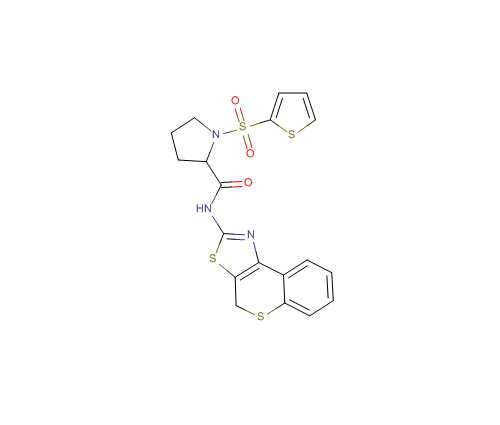 | TDR83016 | 1.88 | 3.71 | 0.5 | 0.45 | 4.5 | 100 | 100 | 0 | 100 | - |
| 7 | 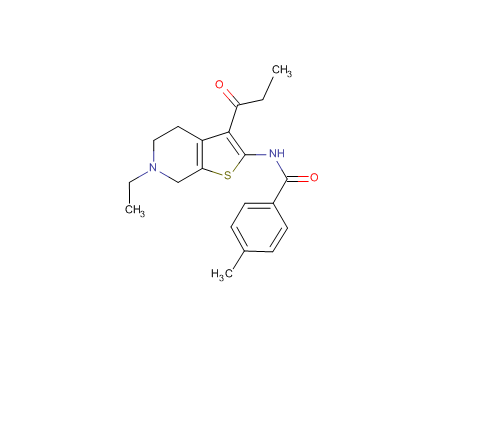 | TDR76871 | >11.79 | 1.87 | 0.48 | 0.32 | 2.32 | 0 | 33.3 | 22.3 | 0 | - |
| 8 | 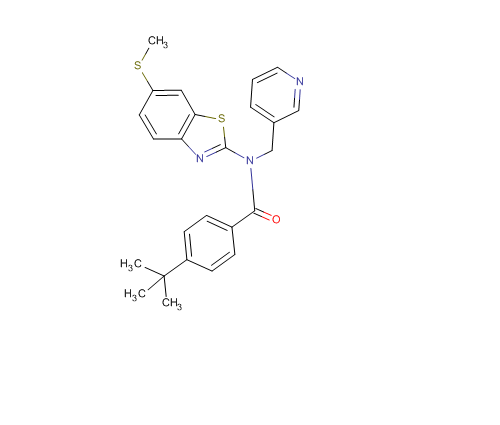 | TDR84420 | 7.16 | 0.15 | 0.56 | >13.43 | >13.43 | 0 | 0 | - | 25 | - |
| 9 | 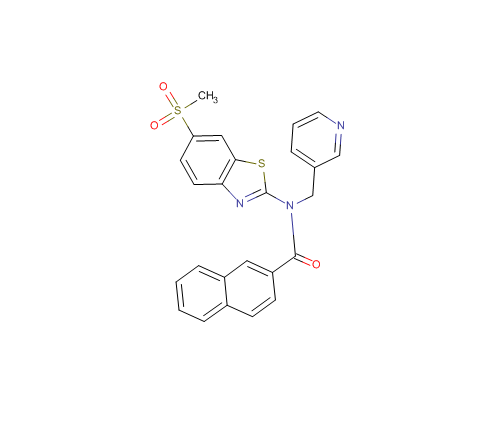 | TDR84431 | >14.21 | >14.21 | 0.23 | >14.21 | 4.6 | 0 | 0 | - | 0 | - |
| 10 | 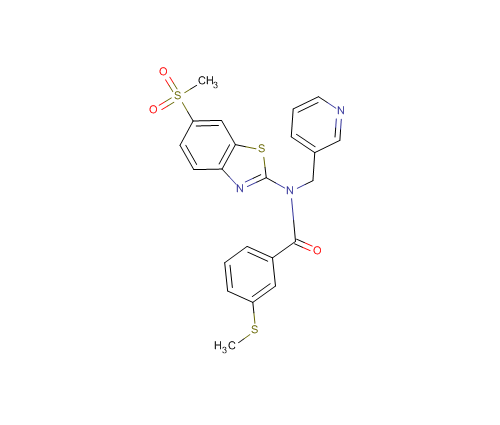 | TDR84433 | >14.09 | 8.28 | 0.38 | >14.09 | >14.09 | 0 | 0 | - | 0 | - |
| 11 | 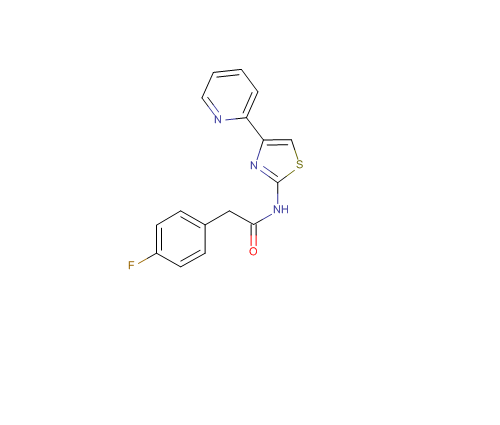 | TDR82963 | >9.4 | 2.97 | 0.33 | 0.31 | >9.4 | 0 | 0 | - | 23 | - |
| 12 | 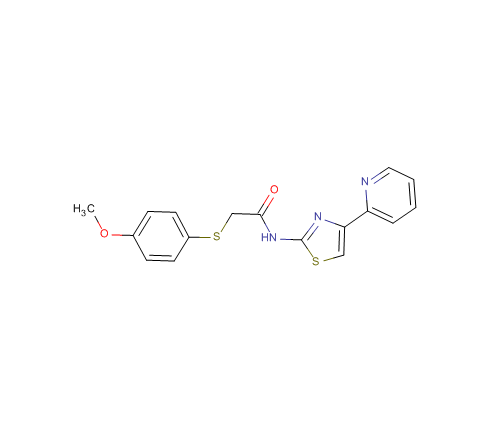 | TDR83845 | >10.72 | >10.72 | 0.36 | 0.35 | >10.72 | 0 | 0 | - | 86 | - |
| 13 | 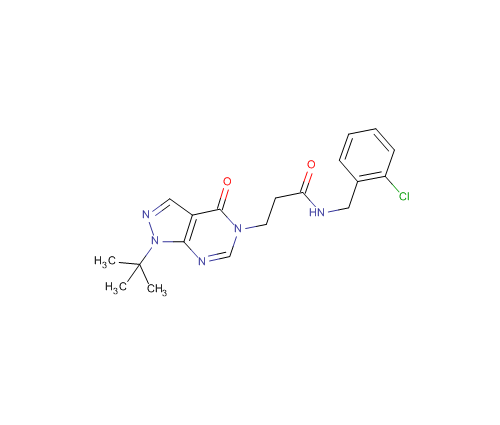 | TDR84851 | >11.64 | >11.64 | >11.64 | >11.64 | >11.64 | 100 | 20 | - | 0 | - |
| 14 | 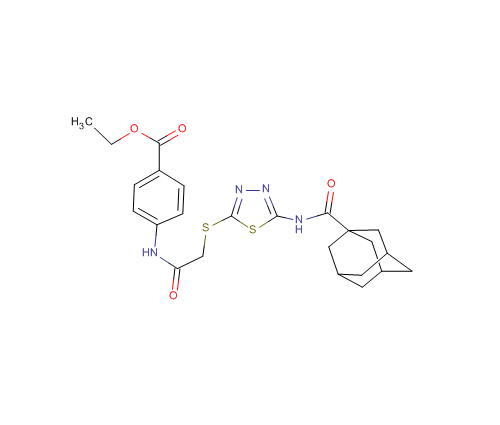 | TDR76705 | >15.02 | >15.02 | >15.02 | >15.02 | >15.02 | 0 | 100 | 100 | 33.3 | - |
| 15 | 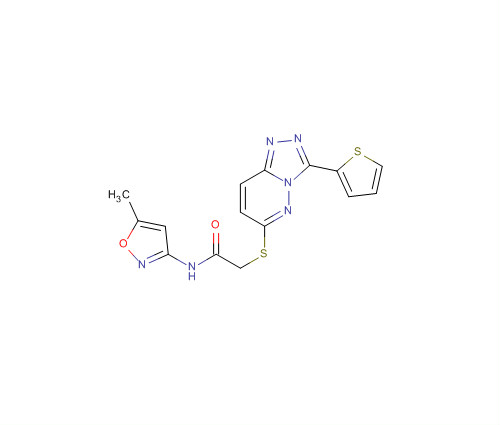 | TDR78826 | >11.17 | 0.16 | 4.97 | 11.17 | 4.29 | 0 | - | - | 6.7 | 0 |
| 16 | 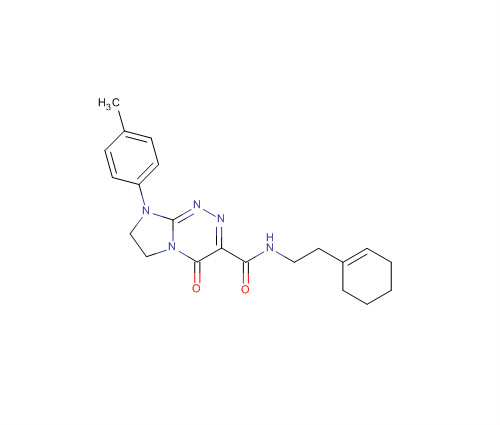 | TDR81281 | >11.38 | 0.15 | >11.38 | >11.38 | 3.69 | 0 | - | - | 0 | - |
| 17 | 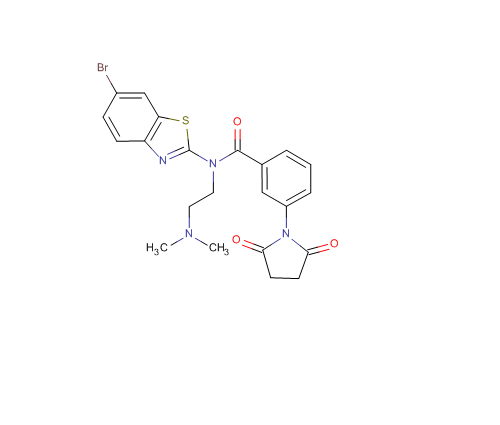 | TDR79337 | >16.14 | 1.45 | 4.94 | 0.36 | 5.1 | 33 | - | 0 | 0 | - |
| 18 | 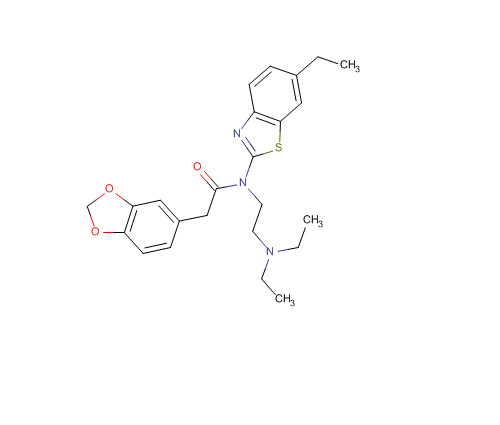 | TDR80505 | >14.28 | 4.52 | 3.61 | 0.46 | 7.4 | 0 | - | - | 0 | - |
| 19 | 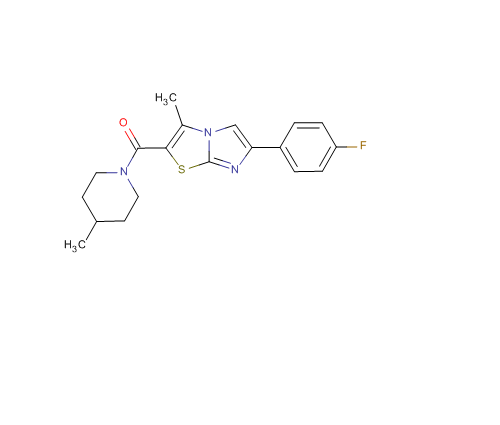 | TDR77657 | >10.72 | >10.72 | >10.72 | 0.42 | >10.72 | 0 | 0 | - | 0 | - |
| 20 | 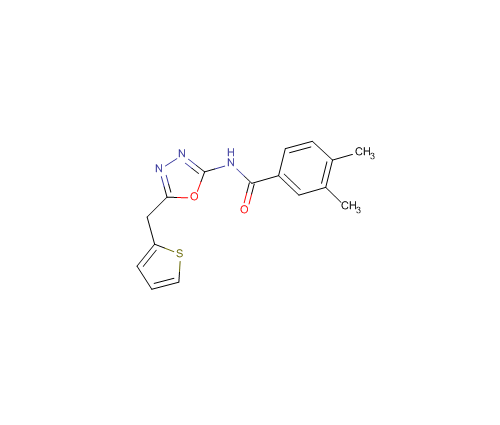 | TDR85275 | 9.4 | 9.4 | 3.01 | 0.38 | 9.4 | 25 | 50 | - | 0 | - |
| 21 | 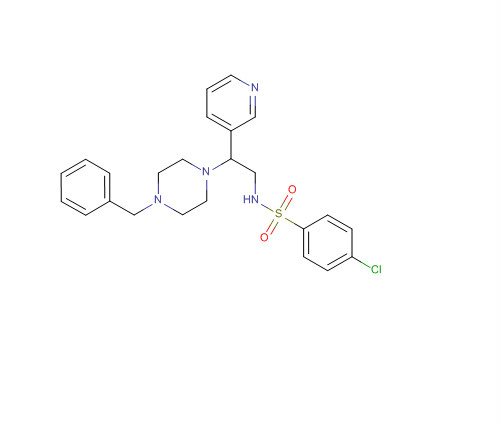 | TDR78023 | >14.13 | 2.18 | 0.63 | >14.13 | >14.13 | 0 | - | - | 0 | - |
| 22 | 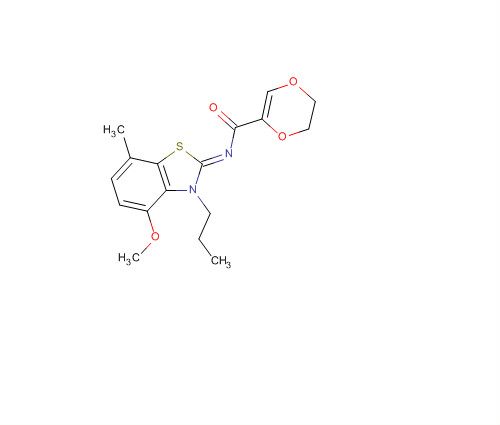 | TDR82272 | >10.45 | 3.18 | 0.89 | >10.45 | >10.45 | 0 | 0 | - | 20 | - |
| 23 | 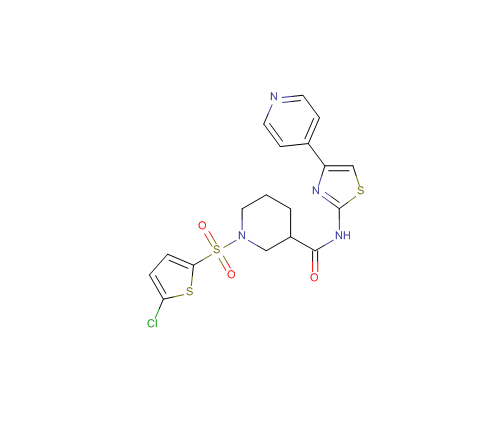 | TDR80165 | >14.07 | 6.68 | 0.24 | 4.5 | 4.56 | 0 | - | - | 50 | - |
| 24 | 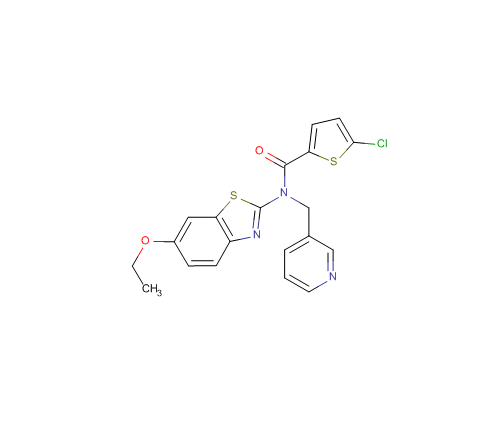 | TDR81636 | >12.2 | 3.55 | 0.66 | 3.52 | 12.2 | 0 | 0 | - | 11 | - |
| 25 | 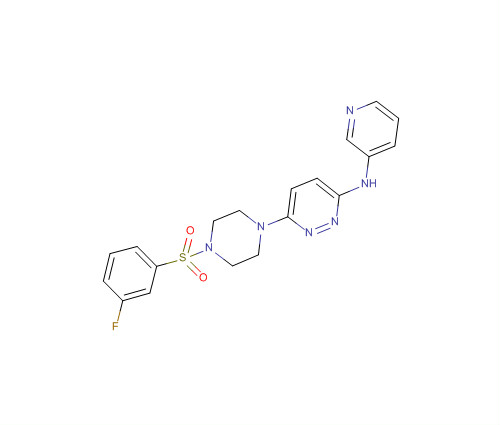 | TDR86129 | 0.94 | 5.44 | >12.43 | >12.43 | 0.4 | 0 | 0 | - | 0 | - |
| 26 | 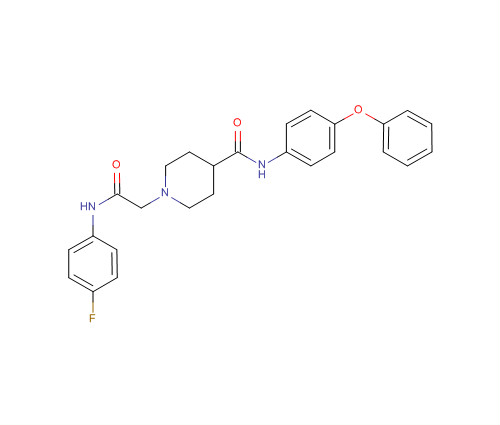 | TDR80731 | >13.43 | >13.43 | >13.43 | >13.43 | 0.7 | 0 | - | - | 0 | - |
| 27 | 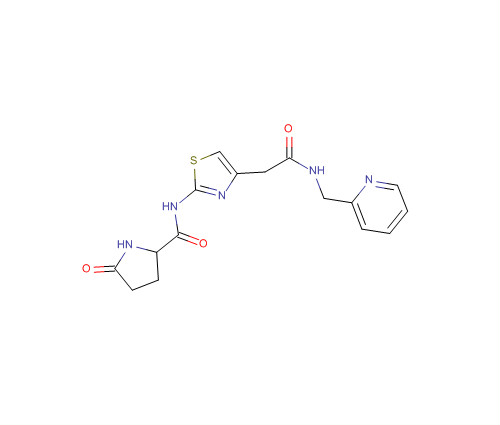 | TDR84826 | >10.78 | >10.78 | >10.78 | >10.78 | >10.78 | 100 | 20 | - | 50 | - |
| 28 | 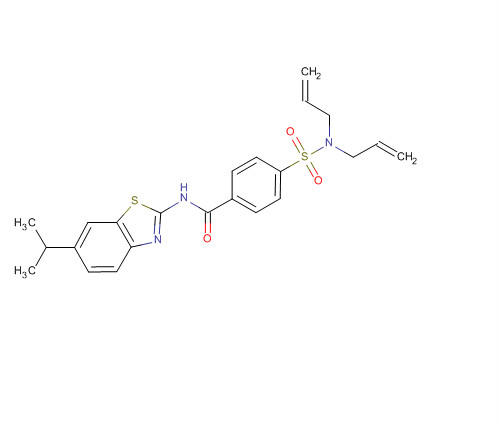 | TDR78794 | >10.99 | >10.99 | 4.48 | >10.99 | >10.99 | 100 | - | - | 0 | - |
| 29 | 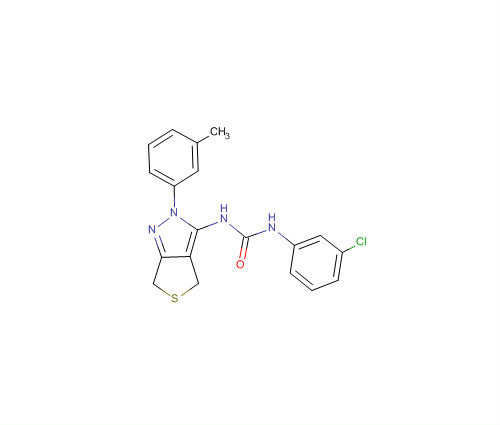 | TDR76699 | >11.55 | >11.55 | 9.48 | >11.55 | 3.75 | 25 | 100 | 100 | 33 | - |
| 30 | 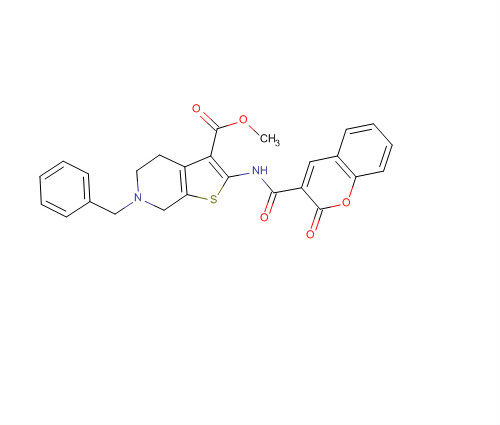 | TDR76873 | 8.92 | >15.33 | 0.64 | >15.33 | 4.96 | 0 | 100 | 100 | 40 | - |
| 31 | 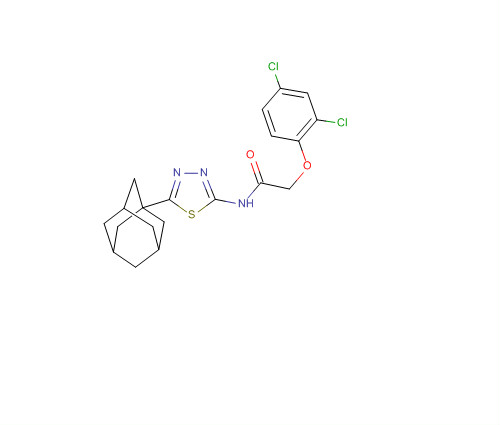 | TDR76660 | >13.15 | >13.15 | >13.15 | >13.15 | 4.26 | 0 | 100 | 0 | 0 | - |
| 32 | 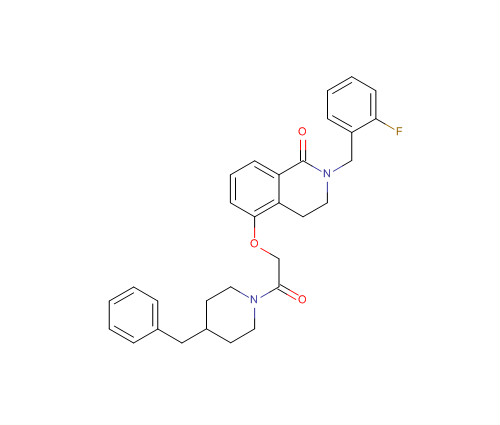 | TDR77144 | 4.56 | 4.54 | 0.56 | 0.47 | 4.73 | 0 | 100 | 100 | 0 | - |
| 33 | 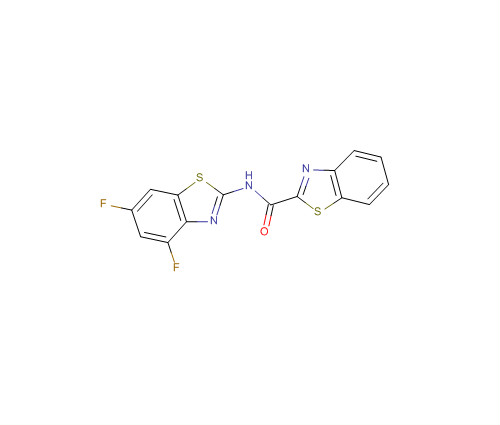 | TDR77323 | >10.42 | >10.42 | >10.42 | >11.12 | >10.42 | 0 | 100 | 50 | - | - |
| 34 | 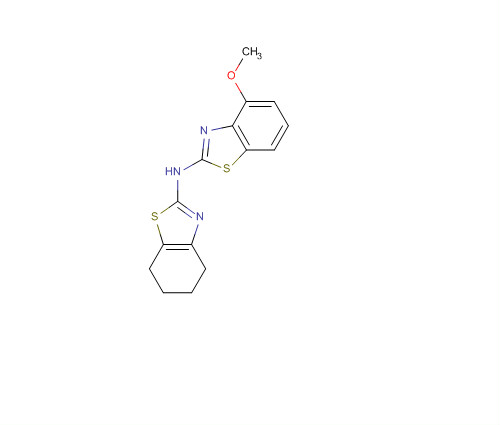 | TDR77577 | >9.52 | >9.52 | 3.81 | >9.52 | >9.52 | 0 | 100 | 50 | - | - |
| 35 | 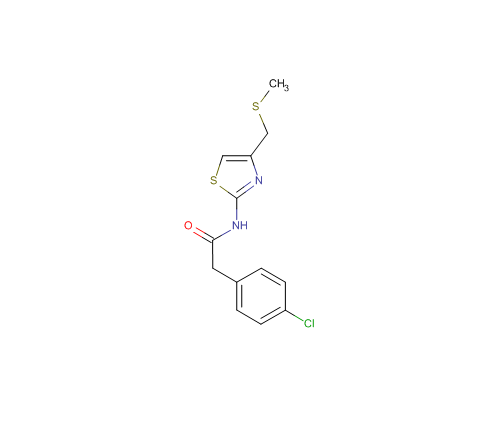 | TDR81206 | 3.7 | 5.98 | 2.47 | 3.0 | >9.38 | 0 | - | - | 100 | 75 |
| 36 | 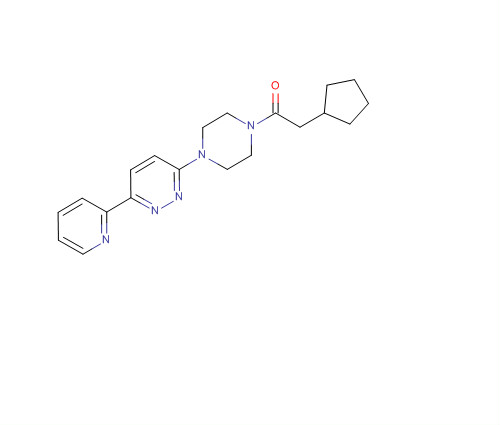 | TDR85084 | 5.57 | 0.73 | 0.47 | 0.41 | 0.34 | 33.3 | 33.3 | - | 0 | - |
| 37 | 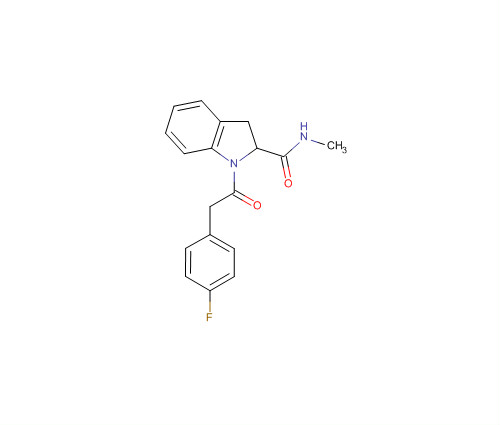 | TDR84116 | >9.37 | >9.37 | >9.37 | 0.08 | >9.37 | 0 | 0 | - | 0 | - |
| 38 | 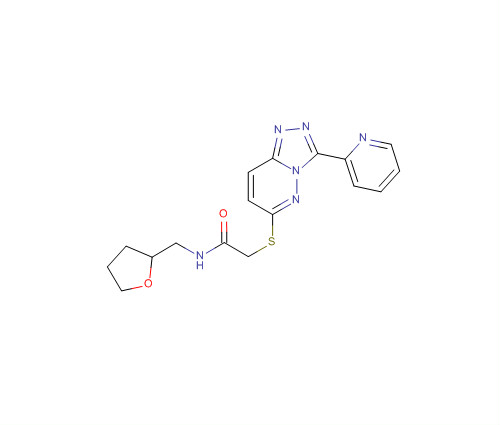 | TDR78834 | >12.1 | 3.15 | 5.2 | 3.3 | 0.39 | 0 | - | - | 0 | - |
| 39 | 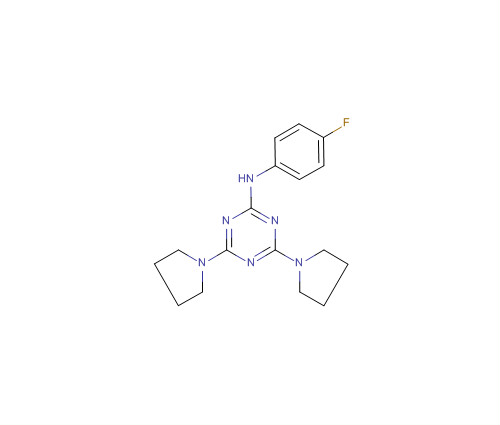 | TDR83682 | >10.95 | >10.95 | >3.8 | 0.44 | >10.95 | 0 | 0 | - | 9 | - |
| 40 | 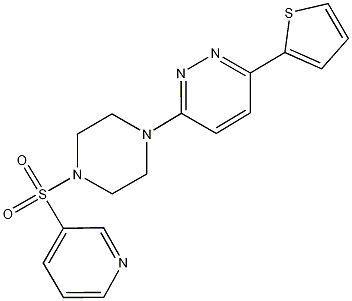 | TDR85115 | >11.6 | >11.6 | >11.6 | >11.6 | 0.38 | 0 | 50 | - | 0 | - |
| 41 | 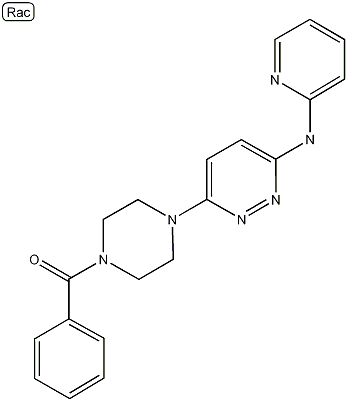 | TDR86082 | >10.8 | >10.8 | >10.8 | >10.8 | 0.35 | 0 | 0 | - | 0 | - |
| 42 | 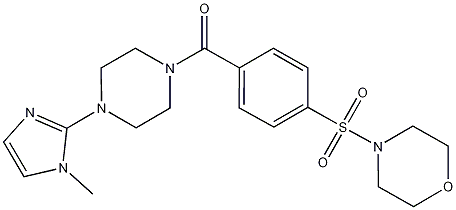 | TDR85459 | >11.4 | >11.4 | >11.4 | >11.4 | 0.37 | 8.3 | 0 | - | 0 | - |
| 43 | 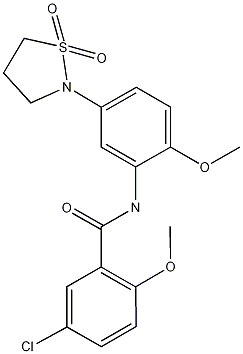 | TDR79456 | >12.3 | >12.3 | 5.0 | 4.3 | 0.4 | 4 | - | - | 13.3 | - |

**Supplementary material legend:**

Supplementary material table:: Case examples of hits identified

Chemical structure numbering from 1 to 39 and TDR ID numbers are used to describe select hit series resulting from the screens from the different pathogens. Only representative case examples are presented. The remaining structures are either made available through www.tdrtargets.org or transferred under legal agreement to partners for further progression. Color coding is the same as in Figure 3 and dashes indicate that results are not available.
